# Dental News Letter—Editorial Change

**Published:** 1853-10

**Authors:** 


### DENTAL NEWS LETTER—EDITORIAL CHANGE.

The October number of this periodical is just at hand, and we are gratified to see the name of J. D. White, M. D., D. D. S., as one of its editors. The News Letter has always been a progressive and advancing periodical, and Dr. White has been one of its ablest contributors. It cannot but be gratifying to the profession to notice the change which a few years has produced in our Dental Periodical Literature. In glancing at those now on our table, we see the oldest dates back some thirteen years. One has just closed its seventh volume, and two are just entering on the same. Independent of the one we control, it affords us much pleasure to notice the continued progress which has been made, and we hazard not much when we say that they compare well with the oldest periodicals of the Medical Profession. The stigma that our profession is not a literary one is fast passing away. There is five times as much written now as ten or fifteen years ago, and twice as well written. The profession should know, however, that those who edit these periodicals have all something else to do also, and we fear the time will be long before it will be otherwise, and we believe it best that they should generally be practical men, yet having much else to do, they cannot be always as exact and particular in correcting and proof-reading as their contributors might like. These should always so carefully write their manuscripts, that the request to cut, and trim, and re-write, would be unnecessary. It is a lamentable fact, that many of our best writers, in point of literary merit, use many hieroglyphics, which the poor editor or compositor has to decipher. When these are from a new hand at the business, it often takes much figuring and a lengthy consultation of foreman, compositors and editor to unravel. To these arduous duties we welcome the senior editor of the News Letter, and at tne next Railroad festival given to editors, we hope to meet the entire editorial corps of the Dental Press.

